# Elevated depressive symptoms and adolescent injury: examining associations by injury frequency, injury type, and gender

**DOI:** 10.1186/1471-2458-14-190

**Published:** 2014-02-21

**Authors:** Mark Asbridge, Sunday Azagba, Donald B Langille, Daniel Rasic

**Affiliations:** 1 Department of Community Health and Epidemiology, Faculty of Medicine, Dalhousie University, Halifax, Nova Scotia, Canada; 2 Department of Psychiatry, Faculty of Medicine, Dalhousie University, Halifax, Nova Scotia, Canada

**Keywords:** Adolescents, Depression, Injury, Violence, Transportation, Unintentional, Gender

## Abstract

**Background:**

Key risk factors for adolescent injury have been well documented, and include structural, behavioural, and psychosocial indicators. While psychiatric distress has been associated with suicidal behaviour and related self-harm, very little research has examined the role of depression in shaping adolescent injury. This study examines the association of elevated depressive symptoms with injury, including total number of injuries and injury type. Gender differences are also considered.

**Methods:**

Data were drawn in 2010–11 from a representative sample of 2,989 high school students (14 to18 years of age) from Nova Scotia, Canada. Self-reported injury outcomes were examined using the 17-item Adolescent Injury Checklist, which captures past six-month injuries. Elevated depressive symptoms were assessed using the Centers for Epidemiological Studies Depression scale. Associations of elevated depressive symptoms with total number of injuries were estimated with negative binomial regression, while associations with specific injury types were estimated with logistic regression. Analyses were conducted in 2012.

**Results:**

Adolescents with elevated depressive symptoms experienced a 40% increase in the total number of injury events occurring in the past six months. The association of elevated depressive symptoms with injury was consistent across injury type; violence-related (OR 2.21, 95% CI 1.61 to 3.03), transport-related (OR 1.53, 95% CI 1.10 to 2.13), and unintentional injuries (OR 1.65, 95% CI 1.20 to 2.27). Gender differences were also observed.

**Conclusion:**

Elevated depressive symptoms play a role in shaping adolescent injury. Interventions aimed at reducing adolescent injury should look to minimize psychosocial antecedents, such as poor mental health, that put adolescents at an elevated risk.

## Background

Injury is the leading cause of death for adolescents in Canada, associated hospitalizations and emergency department visits, [1,2] with Nova Scotia reporting higher rates of unintentional injuries relative to the rest of Canada [3]. Considerable research has gone into understanding risk and protective factors specific to injuries. Evidence has established the associations of certain social determinants with injury, [4-6] particularly among adults, such as gender, [7-9] socio-economic status, [7,8,10,11] rural/urban living, [12] and ethnicity and culture [13,14]. For instance, gender differences persist in injury rates with males being more likely than females to suffer serious injury, [7,8] suggesting male risk-taking propensity and participation in contact sport are possible explanations [9,10,15]. Among adolescents, however, female injury rates are often higher than males, particularly for suicide and self-harm related injury [9]. Similarly, ecological studies suggest that low socioeconomic status and neighbourhood disadvantage lead to higher rates of unintentional injury hospitalization [7,8]. Injury has also been linked with psychosocial risk factors, including alcohol and drug use [16], both of which increase the risk of unintentional (e.g. falls) and intentional injury (traffic crashes, violence and self-harm) [9,17-20].

Outside of suicidal behaviour (non-suicidal self-harm, suicide attempts), the role of mental health in adolescent injury has received very limited attention. Annually, in Canada, 5% of male youth and 12% of female youth, between the ages of 12 and 19, report having experienced a major depressive episode, and depression is the second highest (behind injury) hospital care expenditure among youth [21]. Student surveys from Nova Scotia have found that 20.1% of high school students showed a somewhat elevated risk of depression, with 5.9% showing very elevated depression risk [22].

There is some evidence of an association between depression and injury occurrence among adults. Depression has been linked with suicide and self-harm, [23-25] while serious physical injury has been associated with the subsequent emergence of depression [26,27]. Depression is also frequently comorbid with substance use [28] and conduct problems, [29] each of which increases the risk of acute injury [30,31]. Indirectly, studies of psychological distress, which includes depression subscales, have shown mixed associations with motor vehicle collisions involvement, [32,33] while the reciprocal link between depression and exposure to violence is quite robust [34,35]. The extent to which depression shapes these injuries among young people and with respect to other injury events remains less clear and is an important area of inquiry.

### Purpose

Employing a comprehensive measure of adolescent injury events, [36] the current study looks to examine the association of elevated depressive symptoms and involvement in injury among junior and senior school students (13 to 18 years of age) living in Nova Scotia, Canada. This study addresses two questions: 1) Are elevated depressive symptoms independently associated with injury involvement; and, if so, 2) Does the association persist across different types of injury (intentional and unintentional); and 3) for male and female students? A fuller exploration of these issues will help to inform program and policy efforts to prevent adolescent injury, in terms of addressing commodities, such as depression and anxiety that shape injury patterns and rates.

## Methods

### Data

The 2010–2011 Health Behaviour Survey (HBS) is a representative sample of high school students in the province of Nova Scotia, Canada. Nova Scotia is the most populated province in Atlantic Canada, with almost 1 million people [37]. The province is predominantly English speaking. The HBS was implemented as part of Health Canada’s 2010-2011Youth Smoking Survey (YSS), and drew on the YSS sampling frame. A detailed description about the design and procedure of the YSS has been documented elsewhere [38]. A total of eight secondary schools agreed to participate in the HBS project out of the 10 schools in Nova Scotia that took part in the YSS. All students in grades 10–12 (age 14 to 18) in each participating school were eligible to take part in the survey. A total of 2,989 students participated in the HBS survey and provided completed questionnaires, with a response rate of 57%. Parental consent and individual consent was obtained from all participating students. Data were collected between November 2010 and May 2011. All protocol and materials for both the YSS and HBS received ethics approval from the University of Waterloo and Dalhousie University.

### Measures

#### Dependent variables

*Adolescent injury checklist (AIC):* self-reported injury outcomes were examined using the 17-item AIC, [36] with the wording for some questions slightly modified to reflect local/national jargon. The AIC records types of injuries that are common among adolescents (see the Appendix for a list of the AIC items). Participants were asked if they had been injured in various situations in the past 6 months, such as “By being in a physical fight with someone?”, “By a BB gun, pellet gun or regular gun?”, “By being stabbed?”, “While driving a car, truck or bus?”, “While riding a motorcycle, moped, snowmobile or all-terrain vehicle?”. All questions had response choices of “yes” or “no” and where included as dichotomous (1/0) measures. A total score was derived by summing all 17 individual injury items. The Kuder-Richardson 20 (KR20) coefficient for the 17-item scale was 0.79; this is similar to the alpha coefficient reported in an Australian study, 0.76 [39] and a US study, 0.68 [36]. Sub-scales from the AIC were created with a particular emphasis on violence-related injuries (4-items KR20 = 0.66), transportation-related injury (4-items; KR20 = 0.61), and unintentional injuries (9-items; KR20 = 0.64), where respondents had to have reported at least one injury of that type in the past six months.

#### Independent variables

Elevated depressive symptoms were measured by an 8-item version of the Centers for Epidemiological Studies Depression (CES-D) Scale (range, 0 to 24, Cronbach’s alpha for this sample = 0.91) with a higher score meaning increased depressive symptoms. These items measured feelings over the past week including: “I felt sad”, “I felt depressed”, “I thought my life had been a failure”, “I felt fearful”, “my sleep was restless”, “I felt lonely”, “I had crying spells”, and “I felt that I could not shake off the blues even with help from my family and friends”. Each item was scored on a 4-point Likert-type scale ranging from rarely/never to very often and these items were summed to create a depression score. The depression score was dichotomized at a cut point of 7 or above to indicate a higher risk of being depressed [40]. This 8-item version of the CES-D has been used previously to assess depressive symptoms in adolescents, [41,42] and results are comparable to prevalence estimates in adolescents using the full 20-item CES-D and a 12- item versions [43,44]. While the CES-D measures elevated depressive symptoms in the past week, assessments of test-retest reliability show moderate to strong correlations at three and six months after initial testing [45,46].

Consistent with previous injury studies [7-14], a number of covariates were included that have shown to be associated with injury among adolescents. These include sociodemographic indicators: gender (1 = male, 0 = female); school grade level (10, 11, and 12), weekly spending money ($40 or more, unknown, less than $40); risk-taking indicators: being a current smoker (including daily and occasional smoker); past year heavy drinking (reported drinking ≥5 on one occasion at least 12 times in the last year, compared to drank ≥5 on one occasion fewer than 12 times in the last year, and no drinking in past year); past year marijuana use (used marijuana in the last year frequently (at least 12 times), infrequent marijuana use and no marijuana use) and sexual risk behaviours (reported having multiple sexual partners, single sexual partner and sexually inactive); and individual school indicators: school absence/skipping (3 days or more, 1 or 2 days, no absence from school); academic mark (grade average ≥70% vs. <70%) and participation in one or more school team sports.

### Statistical analysis

To address our primary research question, a multivariable count regression model was estimated to examine the cross-sectional associations between total number of injuries and elevated depressive symptoms. Specifically, a negative binomial count model was estimated due to overdispersion of total injuries. In answering our second question, separate multivariable logistic regression analyses were performed to examine the associations between experiencing different types of injury (using the subscales for violence-related injury, transportation-related injury and unintentional injury) and elevated depressive symptoms. Given the collinearity between alcohol consumption and marijuana use in our data, two models were examined: Model 1 includes all covariates without marijuana use while Model 2 includes marijuana in addition to other covariates with the exception of alcohol consumption. Survey weights were used in all analyses to produce population estimates and adjust for both the unequal probability of selection and student non-response. Some cases were dropped due to missing data giving us a final analytic sample of 2781. All analyses were completed in 2012 using Stata 12 [47].

## Results

Table 1 presents weighted demographic sample characteristics. Of the students in grades 10 to 12, about 51% were male, 14% were current smokers (17% males and 11% females) and 37% were involved in heavy drinking in the past year. Overall, 78% of high school students experienced at least one injury in the past six months, an average of 2.3 injuries, with male students reported a greater number of injuries relative to female students.

**Table 1:** Weighted sample characteristics (%)

**Variable**	**Total**	**Males**	**Females**
Dependent variable			
Adolescent injury checklist score^a^	2.3	2.5	2.1
Violence-related injury	25.0	25.0	12.4
Transport-related injury	26.3	26.3	10.5
Unintentional injury	74.8	74.8	77.6
Independent variable			
Higher risk of depression	23.5	16.3	30.8
Lower risk of depression	76.5	83.6	69.2
Grade level			
10	32.6	33.2	32.0
11	34.4	34.4	34.4
12	33.0	32.4	33.5
Weekly spending money			
$40 or more	36.7	37.9	35.5
Below $40	51.1	50.7	51.5
Unknown	12.2	11.4	12.9
Risk taking behaviours			
Current smoker	13.8	16.7	10.7
Nonsmoker	86.2	83.3	89.3
Heavy drinking	37.4	40.8	34.0
Less heavy drinking	35.3	30.2	40.7
No drinking	27.2	29.0	25.3
Frequent marijuana use	26.3	31.2	21.2
Infrequent marijuana use	18.4	19.0	17.8
No marijuana	55.2	49.8	60.9
Multiple sexual partners	21.2	23.4	19.0
Single sexual partner	27.1	25.5	28.9
Sexually inactive	51.6	51.4	52.1
Individual School measure			
Academic marks 70 & above	74.7	68.3	81.4
Academic marks below 70	25.3	31.7	18.6
Absence from school 3 days or more	21.1	25.2	16.8
Absence from school 1 or 2 days	21.5	20.2	22.8
No absence from school	57.4	54.6	60.4
School team sports	37.4	40.1	34.6
No school team sports	62.6	59.9	65.4
Observations (N)	2,781	1,300	1,481

^a^Adolescent injury checklist is a count variable, the values represents the mean score.

Some variation was observed by injury type. The prevalence of having at least one violence-related injury (25% for males and 12% for females) and transport-related injury (26.3% for males, 11% for females 11%) in the past six months was higher for males than females. Conversely, past six month involvement in unintentional injuries was slightly higher for female students (77.6% vs. 74.8%). Almost one-in-four students had elevated depressive symptoms, with rates for female students (31%) nearly twice that of male students (16%).

### Total number of injuries (AIC score)

The crude incidence-rate ratio (IRR) from the negative binomial regression model (not shown) indicates that those with elevated depressive symptoms had 48% more injuries in the past six months (IRR 1.48, 95% CI: 132, 165). The multivariable analyses (Table 2) confirm a positive association between total injuries and elevated depressive symptoms.

**Table 2:** Multivariable Negative Binomial Regression of total number of injuries on depression and covariates (IRR and 95% CIs reported)

**Variable**	**Model 1**	**95% CI**	**Model 2**	**95% CI**
Higher risk of depression	1.41***	1.26, 1.57	1.42***	1.27, 1.58
Lower risk of depression	Ref	Ref	Ref	Ref
Grade level				
10	1.24***	1.11, 1.39	1.20***	1.08, 1.35
11	1.10*	0.99, 1.22	1.08	0.97, 1.20
12	Ref		Ref	
Gender				
Male	1.16***	1.06, 1.26	1.14***	1.04, 1.25
Female	Ref		Ref	
Weekly spending money, $				
40 or more	1.07	0.97, 1.18	1.07	0.99, 1.20
Unknown	1.06	0.93, 1.20	1.07	0.93, 1.21
Below 40	Ref		Ref	
Risk taking behaviours				
Current smoker	1.20**	1.04, 1.39	1.17**	1.01, 1.35
Nonsmoker	Ref		Ref	
Heavy drinking	1.25***	1.09, 1.42		
Light drinking	1.08	0.96, 1.23		
No drink	Ref			
Frequent marijuana use			1.34***	1.18, 1.53
Infrequent marijuana use			1.13**	1.01, 1.28
No marijuana			Ref	
Multiple partners	1.54***	1.33, 1.78	1.46***	1.26, 1.69
Single partner	1.19***	1.07, 1.32	1.18***	1.07, 1.30
Sexually inactive	Ref		Ref	
Individual school measures				
Academic Marks ≥70%	1.00	0.89, 1.12	1.02	0.92, 1.14
Academic Marks <70%	Ref		Ref	
Absence from school 3 days or more	1.16**	1.01, 1.32	1.14	1.00, 1.29
Absence from school 1 or 2 days	1.053	0.95, 1.17	1.05	0.95, 1.17
No absence from school	Ref		Ref	
School team sports	1.22***	1.11, 1.34	1.25***	1.14, 1.36
No school team sports	Ref		Ref	

***p < 0.01, **p < 0.05.

Compared to those with minimal depressive symptoms, adolescents with elevated depressive symptoms (IRR 1.41, 95% CI: 1.26, 1.57, Model 1) had 41% more injuries in the past six months. Significant differences in the total number of injuries by covariates were observed: students in grade 10 had 24% (IRR 1.24, 95% CI: 1.11, 1.39) more injuries compared to those in grade 12, while there were positive associations between total number of injuries and being a current smoker (IRR 1.20, 95% CI: 1.04, 1.39), heavy drinking (IRR 1.25, 95% CI: 1.09, 1.42), marijuana use (frequent use: IRR 1.34, 95% CI: 1.18, 1.53; infrequent use: IRR 1.13, 95% CI: 1.01, 1.28, Model 2), sexual activity (multiple sex partners: IRR 1.54, 95% CI: 1.33, 1.78; single sex partner: IRR 1.19, 95% CI: 1.07, 1.32), skipping school 3 days or more (IRR 1.16, 95% CI: 1.01, 1.32) and participation in school team sports (IRR 1.22, 95% CI: 1.11, 1.34). Males had 16% more injuries in the past 6 months (IRR 1.16, 95% CI: 1.06, 1.26) compared to females.

### Violence-related injury

Crude logistic regression results indicate that elevated symptoms of depression were associated with increased odds of experiencing a violence-related injury (Odds Ratio [OR] 2.08, 95% CI: 1.60, 2.71). Multivariable logistic regression results are reported in Table 3 and show that having elevated depressive symptoms increased the odds of having experienced a violence-related injury in the past month (OR 2.21, 95% CI: 1.61, 3.03, Model 1). Also students who: were in grade 10, male, currently smoked, were involved in past year heavy drinking, frequently used marijuana in the past year, were sexually active, had multiple sex partners, and skipped school had an increased odds of reporting a violence-related injury in the past 6 months.

**Table 3:** Multivariable Logistic Regression of violence-related injury on depression and covariates (OR and 95% CIs reported)

**Variables**	**Model 1**	**95% CI**	**Model 2**	**95% CI**
Higher risk of depression	2.21***	1.61, 3.03	2.25***	1.64, 3.10
Lower risk of depression	Ref	Ref	Ref	Ref
Grade level				
10	2.04***	1.46, 2.85	1.89***	1.35, 2.64
11	1.27	0.90, 1.78	1.32	0.93, 1.85
12	Ref		Ref	
Gender				
Male	2.53***	1.89, 3.37	2.40***	1.80, 3.20
Female	Ref		Ref	
Weekly spending money, $				
40 or more	1.10	0.82, 1.48	1.16	0.86, 1.56
Unknown	0.69	0.44, 1.08	0.77	0.49, 1.21
Below 40	Ref		Ref	
Risk taking behaviours				
Current smoker	2.20***	1.54, 3.14	1.95***	1.35, 2.81
Nonsmoker	Ref		Ref	
Heavy drinking	1.61**	1.06, 2.44		
Light drinking	1.36	0.93, 2.00		
No drink	Ref			
Frequent marijuana use			2.22***	1.53, 3.23
Infrequent marijuana use			1.11	0.75, 1.66
No marijuana			Ref	
Multiple partners	2.48***	1.69, 3.65	2.27***	1.52, 3.37
Single partner	1.44**	1.01, 2.06	1.38	0.97, 1.97
Sexually inactive	Ref		Ref	
Individual school measures				
Academic Marks ≥70%	0.83	0.61, 1.14	0.89	0.65, 1.21
Academic Marks <70%	Ref		Ref	
Absence from school 3 days or more	1.69***	1.18, 2.42	1.52**	1.05, 2.18
Absence from school 1 or 2 days	1.63***	1.17, 2.28	1.54**	1.10, 2.14
No absence from school	Ref		Ref	
School team sports	0.98	0.74, 1.31	1.03	0.77, 1.38
No school team sports	Ref		Ref	

***p < 0.01, **p < 0.05.

### Transport-related injury

Crude logistic regression results indicate that elevated symptoms of depression were associated with increased odds of experiencing a transport-related injury (OR 1.38. 95% CI: 1.05, 1.82). Multivariable logistic regression results for transport-related injury are reported in Table 4. Again, having elevated depressive symptoms (OR 1.53, 95% CI: 1.10, 2.13) was associated with having experienced a transport-related injury in the past 6 months. Increased odds of reporting transport-related were observed for: males, those who were sexually active, and those who skipped school 3 days or more. Higher academic performance was protective of transport-related injury.

**Table 4:** Multivariable Logistic Regression of transport-related injury on depression and covariates (OR and 95% CIs reported)

**Variables**	**Model 1**	**95% CI**	**Model 2**	**95% CI**
Higher risk of depression	1.53**	1.10, 2.13	1.50**	1.07, 2.09
Lower risk of depression	Ref	Ref	Ref	Ref
Grade level				
10	1.45**	1.03, 2.04	1.44**	1.02, 2.04
11	1.16	0.82, 1.62	1.17	0.84, 1.65
12	Ref		Ref	
Gender				
Male	3.11***	2.29, 4.23	3.07***	2.26, 4.17
Female	Ref		Ref	
Weekly spending money, $				
40 or more	0.96	0.70, 1.32	0.95	0.69, 1.31
Unknown	1.04	0.69, 1.58	1.15	0.76, 1.74
Below 40	Ref		Ref	
Risk taking behaviours				
Current smoker	1.16	0.79, 1.70	1.20	0.82, 1.77
Nonsmoker	Ref		Ref	
Heavy drinking	1.14	0.77, 1.70		
Light drinking	0.86	0.58, 1.27		
No drink	Ref			
Frequent marijuana use			1.34	0.94, 1.91
Infrequent marijuana use			1.23	0.84, 1.79
No marijuana			Ref	
Multiple partners	2.43***	1.65, 3.58	2.23***	1.50, 3.31
Single partner	1.65***	1.18, 2.31	1.56***	1.12, 2.18
Sexually inactive	Ref		Ref	
Individual school measures				
Academic Marks ≥70%	0.68**	0.50, 0.92	0.71**	0.52, 0.96
Academic Marks <70%	Ref		Ref	
Absence from school 3 days or more	1.51**	1.06, 2.17	1.60**	1.11, 2.30
Absence from school 1 or 2 days	0.91	0.64, 1.29	0.94	0.66, 1.34
No absence from school	Ref		Ref	
School team sports	1.18	0.89, 1.57	1.19	0.89, 1.59
No school team sports	Ref		Ref	

***p < 0.01, **p < 0.05.

### Unintentional injury

Crude logistic regression results indicate that elevated symptoms of depression were associated with increased odds of experiencing an unintentional injury (OR 1.59, 95% CI: 1.20, 2.11). The multivariable results for unintentional injury are presented in Table 5. As above, elevated depressive symptoms was associated with having experienced at least one unintentional injury in the past 6 months (OR 1.65, 95% CI: 1.20, 2.27). Positive significant associations were also found between unintentional injury and heavy drinking, marijuana use, being sexually active, and participation in school team sports.

**Table 5:** Multivariable Logistic Regression of unintentional injury on depression and covariates (OR and 95% CIs reported)

**Variables**	**Model 1**	**95% CI**	**Model 2**	**95% CI**
Higher risk of depression	1.65***	1.20, 2.27	1.66***	1.21, 2.28
Lower risk of depression	Ref	Ref	Ref	Ref
Grade level				
10	1.26	0.92, 1.72	1.15	0.84, 1.57
11	1.01	0.75, 1.38	0.93	0.68, 1.27
12	Ref		Ref	
Gender				
Male	0.82	0.64, 1.06	0.82	0.64, 1.06
Female	Ref		Ref	
Weekly spending money, $				
40 or more	1.17	0.88, 1.56	1.22	0.92, 1.63
Unknown	1.03	0.72, 1.47	1.01	0.70, 1.47
Below 40	Ref		Ref	
Risk taking behaviours				
Current smoker	0.86	0.56, 1.31	0.78	0.50, 1.21
Nonsmoker	Ref		Ref	
Heavy drinking	1.94***	1.38, 2.72		
Light drinking	1.26	0.93, 1.69		
No drink	Ref			
Frequent marijuana use			1.83***	1.24, 2.72
Infrequent marijuana use			1.44**	1.00, 2.08
No marijuana			Ref	
Multiple partners	1.60**	1.09, 2.34	1.50**	1.02, 2.22
Single partner	1.59***	1.18, 2.14	1.64***	1.22, 2.20
Sexually inactive	Ref		Ref	
Individual school measures				
Academic Marks ≥70%	1.12	0.83, 1.49	1.17	0.87, 1.57
Academic Marks <70%	Ref		Ref	
Absence from school 3 days or more	1.12	0.79, 1.59	1.12	0.79, 1.59
Absence from school 1 or 2 days	1.20	0.88, 1.63	1.18	0.87, 1.60
No absence from school	Ref		Ref	
School team sports	1.74***	1.33, 2.28	1.84***	1.42, 2.41
No school team sports	Ref		Ref	

***p < 0.01, **p < 0.05.

### Gender stratification and injury

Finally, given gender differences in the prevalence of depression, all models were re-run stratifying on gender. Table 6 presents gender-stratified results and reports only associations between elevated depressive symptoms and injury. In terms of total number of injuries, a positive association with having elevated depressive symptoms was similar for male and female students; however, across injury types gender differences were observed. While having elevated depressive symptoms was positively associated with being involved in a violence-related injury for both male (OR 2.10, 95% CI: 1.31, 3.35) and female (OR 2.43, 95% CI: 1.34, 3.38) students, it was associated with transport-related injuries for males only (OR 1.92, 95% CI: 1.22, 3.03), and unintentional injuries for females only (OR 1.81, 95% CI: 1.22, 2.68).

**Table 6:** Gender stratified multivariable regression estimates for total injuries and injury type (violence-related, transportation-related, and unintentional) on depression and covariates (IRR or OR and 95% CIs)

	**Males**				**Females**			
**Dependent variable**	**Model 1**	**95% CI**	**Model 2**	**95% CI**	**Model 1**	**95% CI**	**Model 2**	**95% CI**
**Total number of injuries**	1.50***	1.24, 1.81	1.51***	1.25, 1.81	1.39***	1.24, 1.55	1.39***	1.24, 1.56
**Violence-related injury**	2.10***	1.31, 3.35	2.13***	1.34, 3.38	2.43***	1.57, 3.78	2.56***	1.63, 4.04
**Transport-related injury**	1.92***	1.22, 3.03	1.85***	1.17, 2.94	1.21	0.78, 1.88	1.18	0.76, 1.84
**Unintentional injury**	1.54	0.91, 2.61	1.58	0.94, 2.66	1.81***	1.22, 2.68	1.71***	1.17, 2.50

All stratified models report adjusted odd ratios with the exception of the AIC estimates, which are adjusted incidence rate ratios.

***p < 0.01.

Model 1 includes all covariates with alcohol use and without marijuana use.

Model 2 includes all covariates with marijuana use and without alcohol use.

## Discussion

Employing the Adolescent Injury Checklist, high school students in Nova Scotia, Canada were found to have substantial injury rates, with 78% of adolescents experiencing at least one injury in the past 6 months, with an average of 2.3 injuries. One-in-four young people reported experiencing at least one intentional injury (violence-related or transport-related) in the past 6 months, while nearly three-quarters of young people were involved in at least one unintentional injury. These injury rates are substantially higher than what is observed in hospitalization data, [48], though comparable to other studies relying on self-reported injury; one key difference is that many other self-report studies focus on injury that required medical attention [10,11,16,17,49-51]. Observed rates, both prevalence and average number of injuries, were very similar to earlier studies using the AIC from the United States and Australia [36,39].

The main objectives of this study were to examine the association of elevated depressive symptoms with involvement in injury, and to assess the consistency of any association across injury type. Clear associations between elevated depressive symptoms and injury were observed; students with elevated depressive symptoms were more likely to have been involved in an injury the preceding six months and had, on average, experienced a greater number of injuries (approximately 40% more injuries). The association of elevated depressive symptoms with injury was also consistent across all three injury types – violence, transport, and unintentional injuries – with the strongest association for violence-related injuries. While evidence of an association with depression has previously been observed for certain injuries, particularly suicide and self-harm, [24,25] this is the first study to report an association with a broad range of injuries, as well as total number of injuries, among young people.

How might these associations be understood? First, core symptoms of elevated depressive symptoms are poor concentration and poor sleep [52]. Cognitive studies have also shown these impairments, particularly in treatment seeking adolescents, where depressed individuals show poor executive function, attention and psychomotor skills [53]. In particular, such impairments would increase risk of unintentional and transport related injuries. However, the association between distress and subsequent motor vehicle crash has not been consistent, [32] suggesting that other explanations are likely involved. Some exposures to violence, vehicle crash or other injuries may be experienced as psychological trauma, which leads to depressive symptoms [54]. Second, elevated depressive symptoms is frequently comorbid with conduct problems [55,56] which can increase the risk of injury, especially through unsafe driving and violence [57]. While some studies have suggested that conduct problems may lead to elevated depressive symptoms, there is also evidence that these associations may be manifestations of broader, underlying problems. Deviant peer affiliations, [58] social disadvantage and parental psychopathology [59] may lead to increases in both depression and risk-behaviours leading to injury.

Interestingly, our findings were further nuanced by gender. While elevated depressive symptoms were associated with total number of injuries and violence-related injuries, the link to transport-related injuries was present only for male adolescents, and the association with unintentional injuries was evident only for female adolescents. Gender differences appear to be a product of both differences in rates of elevated depressive symptoms between male and female students, but also injury patterns. Risky driving and resultant injuries have historically been higher in young males [60]. Young males are more likely to be involved in traffic crashes leading to injury or death, and to more-frequently partake in the risk behaviours that lead to such crashes, including impaired driving, speeding, and aggressive driving [61]. In terms of unintentional injuries, our measure captures being cut, bruised or bleeding, with a non-specific cause, and may be suggestive of self-harm related behaviours or of being the victim of intimate interpersonal violence, [62] which occurs more frequently in females. Gender differences in injury maybe also speak to the differential ways that males and females respond (externalizing or internalizing) to feelings of depression and psychiatric distress [63].

This study is not without limitations. First, it should be noted that the measure of elevated depressive symptoms relates to the previous seven days, while the injury question measures any events in the past six months. Similarly, the data is cross-sectional; as such claims of association, but not causation, can be made about the observed relationships between elevated depressive symptoms and injury. Third, the response rate for the survey was 57% and thus the generalizability of results should be interpreted with caution. While the data were weighted to provide population estimates, those at highest risk of injury and depression were likely under-represented in the survey and thus our estimates of an association are biased downward (conservative). Finally, the survey does not include other key injury determinants, including appropriate measures of socioeconomic status (i.e. family income or relative wealth) and family structure, as well as key confounders, such as impulsivity or risk taking propensity.

## Conclusions

This study, with a representative sample of senior high school students, adds to our understanding of the role of depression in shaping adolescent injury. Interventions aimed at reducing or preventing adolescent injury should aim to modify not only the opportunities, circumstances, or environments in which injuries occur, but look to minimize the psychosocial and behaviour antecedents, such as substance use, depression and anxiety, that put adolescents at an elevated risk. Similarly, from a clinical standpoint, our findings point to a potential opportunity for primary-care physicians who treat young people for injuries to ask about feelings of depression and overall mental health.

## Appendix

Below are some ways that people get hurt or injured. In the past 6 months were you injured by the following (Table 7)?

**Table 7:** Adolescent injury checklist

	**Total (%)**
Violence-related injuries	
By being in a physical fight with someone	11.7
By a BB gun, pellet gun or regular gun	7.6
By being physically attacked	7.2
By being stabbed	3.4
At least one violence-related injury	25.0
Transport-related injuries	
While driving a car, truck or bus	3.8
While riding in a car, truck or bus	4.9
While riding a bicycle, skateboard or rollerblades	12.3
While riding a motorcycle, moped, snowmobile or all-terrain vehicle (ATV)	5.9
At least one transport-related injury	26.3
Unintentional injuries	
By getting cut, bruised, bleeding	60.7
By being hit by something, like a rock or glass	9.2
By nearly drowning	2.1
By falling	33.3
By being burned by fire, chemicals, electricity or hot liquid	20.1
By an animal or serious insect bite	7.4
By a team sport, athletic activity, or exercise	39.7
By being hit by a moving vehicle while walking	3.2
By accidentally drinking or eating a dangerous substance	3.6
At least one unintentional injury	74.8

## Competing interests

The authors declare that they have no competing interests.

## Authors’ contributions

MA conceived of the study, and drafted the manuscript. SA carried out the analysis and edited and revised the manuscript. DL conceived of the study and edited and revised the manuscript. DR edited and revised the manuscript. All authors read and approved the final manuscript.

## Pre-publication history

The pre-publication history for this paper can be accessed here:

http://www.biomedcentral.com/1471-2458/14/190/prepub
